# Immunonutrition for patients undergoing elective surgery for gastrointestinal cancer: impact on hospital costs

**DOI:** 10.1186/1477-7819-10-136

**Published:** 2012-07-06

**Authors:** Josephine A Mauskopf, Sean D Candrilli, Hélène Chevrou-Séverac, Juan B Ochoa

**Affiliations:** 1 RTI Health Solutions, Durham, NC, USA; 2 Nestlé Health Sciences, Vevey, Switzerland; 3 FACS; Nestlé Healthcare Nutrition and University of Pittsburgh, Pittsburgh, PA, USA

## Abstract

**Background:**

Oral or enteral dietary supplementation with arginine, omega 3 fatty acids and nucleotides (known as immunonutrition) significantly improve outcomes in patients undergoing elective surgery. The objective of the study was to determine the impact on hospital costs of immunonutrition formulas used in patients undergoing elective surgery for gastrointestinal cancer.

**Methods:**

US hospital costs of stay with and without surgical infectious complications, and average cost per day in the hospital for patients undergoing elective surgery for gastrointestinal cancer were estimated using data from the Healthcare Cost and Utilization Project’s 2008 Nationwide Inpatient Sample. These costs were then used to estimate the impact of perioperative immunonutrition on hospital costs using estimates of reduction in infectious complications or length of stay from a meta-analysis of clinical trials in patients undergoing elective surgery for gastrointestinal cancer. Sensitivity of the results to changes in baseline complication rates or length of stay was tested.

**Results:**

From the meta-analysis estimates, use of immunonutrition resulted in savings per patient of $3,300 with costs based on reduction in infectious complication rates or $6,000 with costs based on length of hospital stay. Cost savings per patient were present for baseline complication rates above 3.5% or when baseline length of stay and infectious complication rates were reduced to reflect recent US data for those with upper and lower GI elective cancer surgery (range, $1,200 to $6,300).

**Conclusions:**

Use of immunonutrition for patients undergoing elective surgery for gastrointestinal cancer is an effective and cost-saving intervention.

## Background

Infectious and other types of complications following gastrointestinal cancer, head and neck cancer, and cardiac surgery are frequent and add significantly to patient morbidity as well as to hospital length of stay and costs [1,2]. Published estimates of complication rates after surgery for patients with gastrointestinal cancer, head and neck cancer, and cardiac disease suggest that these rates range between 15% and 54% [3-6]. Infectious complications include wound infections, abdominal abscess, pneumonia, urinary tract infections, and sepsis. Other types of complications include anastomotic leaks, acute renal failure, and cardiovascular events. Complications prolong the recovery period for the patient, require additional days in the hospital, may increase mortality and increase hospital costs [1].

Immunonutrition formulas composed of arginine, omega-3 fatty acids, and nucleotides improve host defenses, likely through the provision of key nutrients that maintain T-lymphocyte and other immune functions [5,7]. Immunonutrition has been studied in multiple clinical trials, consistently showing reduced postsurgical infectious complications, and some studies also showing a decrease in selected noninfectious complications in patients undergoing elective surgery, including patients undergoing elective surgical interventions for gastrointestinal cancer. Several meta-analyses have summarized the results of these clinical trials, all demonstrating a 38%–62% risk reduction in infections and other complications [3-9]. In particular, Waitzberg and colleagues [4] examined the relationship between hospital length of stay or postoperative infectious complications or anastomotic leak, a noninfectious complication, and the use of immunonutrition using a nutritionally complete formula containing supraphysiologic quantities of arginine, omega-3 fatty acids, and nucleotides (IMPACT®, Nestlé Science Health, Vevey, Switzerland). The results of the Waitzberg meta-analysis showed that, for all nutrition intervention strategies and for all surgical patients studied, use of immunonutrition resulted in lower postsurgical infectious complications, a lower rate of anastomotic leak, and shorter length of stay.

In this paper we evaluated the impact on per-patient hospital costs arising from the use of immunonutrition perioperatively (both before and after surgery) in patients undergoing elective surgery for gastrointestinal (GI) cancer using the results from Waitzberg and colleagues [4] applying estimates of the cost of care of this patient population derived from the Healthcare Cost and Utilization Project’s (HCUP’s) 2008 Nationwide Inpatient Sample (NIS) [10,11].

## Methods

### Clinical data

Table 1 presents the clinical outcome data used in the study. Waitzberg and colleagues [4] identified 6 perioperative studies in patients undergoing elective upper or lower gastric surgery for gastrointestinal or pancreatic cancer. Patients in the control groups in these studies received enteral feeding with standard isocaloric, isonitrogenous formulas. Patients in the treatment group received IMPACT as immunonutrition support. From each study, for the immunonutrition and control groups, Waitzberg and colleagues [4] extracted data on hospital length of stay and number and type of the following postsurgical infectious complications: wound infection, abdominal abscess, pneumonia, urinary tract infection, and sepsis. Waitzberg and colleagues [4] also extracted data on one noninfectious complication, anastomotic leak. The difference in the mean length of stay and the percentage of patients experiencing one of the abstracted infectious complications in the control and immunonutrition groups were derived from the perioperative immunonutrition support studies of GI surgery patients presented in Waitzberg et al. [4].

**Table 1:** **Perioperative immunonutrition support: clinical outcomes**^**a**^

**Outcome variable**	**With perioperative immunonutrition**^**b**^	**Without immunonutrition**	**Difference or RR (95% CI)**
	**N = 442**	**N = 447**	
Mean length of stay	13.31	15.48	Δ = −2.18
Percentage reduction in length of stay			14.1%
Percentage with any infectious complications	14.71%	31.32%	RR = 0.46
Number (%) in pooled trial populations with specific complications^c^			
Wound infection	26 (5.9%)	43 (9.6%)	RR 0.61
			(0.38, 0.96)
Abdominal abscess	9 (2.0%)	21 (4.7%)	RR 0.43
			(0.21, 0.91)
Pneumonia	25 (5.7%)	47 (10.5%)	RR 0.54
			(0.34, 0.87)
Urinary tract infection	11 (2.5%)	20 (4.5%)	RR 0.53
			(0.23, 1.19)
Sepsis	8 (1.8%)	16 (3.6%)	RR 0.53
			(0.22, 1.27)
Anastomotic leak	15 (3.4%)	29 (6.5%)	RR 0.52
			(0.28, 0.95)

Abbreviations: Δ, absolute difference; *RR* relative risk with 95% confidence interval in ().

^a^ Calculated from data reported in Waitzberg et al. [4].

^b^ Three 240-mL (8-ounce) servings of oral supplement per day for five days prior to surgery, followed by early enteral feeding (within 24 hours postsurgery) at a goal of one liter per day.

^c^ Note that a patient may experience more than one complication.

### Costing methodology

We used two methods to estimate the mean cost of a hospital stay for each group. In the first costing method, we estimated average cost of a hospital stay with infectious complications and average cost of a hospital stay without infectious complications for elective GI cancer surgical patients, using the 2008 HCUP NIS data. We then applied these estimated costs to the number of patients with and without infectious complications in the immunonutrition and control groups to estimate the mean hospital cost per stay for each group. In the second costing method, we multiplied the mean length of stay for each group by the mean cost per day in hospital for all elective GI cancer surgical patients in the 2008 HCUP NIS. The methods for estimating the hospital cost per stay and hospital cost per day used in this analysis are described in detail below.

#### Database description, conversion of charges to costs, and identification of GI cancer surgery patients and infectious complications

Nationally representative inpatient discharge data were used to estimate hospital costs per stay and per day for patients with a diagnosis of gastrointestinal cancer. Data for this analysis were taken from the 2008 HCUP NIS, a stratified sample of hospitals drawn from the subset of hospitals in the United States (US) that make their data available to HCUP [10,11]. The NIS is the largest, all-payer inpatient care database in the US and contains data from approximately eight million hospital stays in 2008. The database contains clinical and resource use information typically included in discharge abstracts, including patient demographics, International Classification of Diseases, 9th Revision, Clinical Modification (ICD-9-CM) diagnosis and procedure codes, length of stay, charges incurred, discharge status, admission type and source, and hospital-specific characteristics. Survey design elements provided with the NIS allow for generating nationally representative estimates. We converted hospital charges to costs by applying facility-specific cost-to-charge ratios, provided by HCUP, and then adjusted all costs to 2010 US dollars using the medical care component of the Consumer Price Index. All analyses were conducted using SAS statistical software (SAS®, Cary, North Carolina, US); survey procedures (that is, PROC SURVEYMEANS and SURVEYREG) were used to account for the complex survey design of the NIS. 

As the NIS contains information on the type of admission (e.g., emergency, urgent, elective) associated with each unique hospital stay, we first identified all inpatient stays that were classified as an elective admission. We next identified stays with evidence of upper or lower gastrointestinal cancer (all subcategories) using specific ICD-9-CM diagnosis codes (150.xx, 151.xx, 152.xx, 153.xx, 154.xx, 157.xx, 158.xx, and 159.xx) that also had upper or lower gastrointestinal surgery as identified by ICD-9-CM procedure codes 42.xx, 43.xx, 45.7x, 45.8, 46.xx, 47.xx, 48.xx, 49.0, 49.1, 49.3 and 52.xx.

ICD-9-CM diagnosis codes were used to identify those patients who experienced one or more of the following infectious complications: wound infection, ICD-9-CM codes 031.xx (diseases due to other mycobacteria) and 039.xx-041.xx (actinomycotic infections, other bacterial diseases, and bacterial infection in conditions classified elsewhere); abdominal abscess, ICD-9-CM codes 567.22 (peritoneal abscess) and 998.59 (other postoperative infection, abscess); pneumonia, ICD-9-CM codes 480.xx-485.xx (viral pneumonia; pneumococcal pneumonia; other bacterial pneumonia; pneumonia due to other specified organism; bronchopneumonia, organism unspecified; and pneumonia, organism unspecified); urinary tract infection, ICD-9-CM code 599.0 (urinary tract infection, unspecified/pyuria); sepsis and septicemia, ICD-9-CM-codes 995.91 (systemic inflammatory response syndrome due to infectious process without organ dysfunction) and 038.xx (infectious organisms in the bloodstream); and anastomotic leak, ICD-9-CM code 997.4 (digestive system complications not elsewhere classified including intestinal internal anastomosis and bypass).

#### Estimation of average cost per day in the hospital and average cost per stay in the Hospital for those with and without complications

To compute the average cost per day in the hospital for the patient population of interest, the cost per stay was divided by the length of stay for each patient, and the mean average cost per day was computed for the patient population of interest, those with an elective admission for upper or lower gastrointestinal cancer surgery. For the sensitivity analyses, average costs per day were also estimated separately for those having upper GI cancer surgery and for those having lower GI cancer surgery. Patients having procedure codes for both upper and lower GI cancer surgery were included in both analyses.

To compute the average cost per stay in the hospital for those with and without complications, the 2008 HCUP NIS patients with elective surgery for GI cancer were subdivided into either those with or without at least one of the ICD-9-CM codes for infectious complications listed above or those with and without infectious or other digestive complications including anastomosis and bypass and the average cost per hospital stay estimated for each population subgroup. These estimates were also generated for the infectious complications subgroups for the upper GI and lower GI populations separately.

#### Estimation of impact of immunonutrition formulas on hospital costs

The cost savings per patient associated with nutrition support with immunonutrition formulas were calculated using two different methods: (1) multiplying the Waitzberg and colleagues [4] estimated reduction in length of stay per patient by the average cost per day in the hospital and (2) using the following formula to estimate the mean hospital cost per patient for each treatment group and computing the difference between the groups: (cost per stay with no infectious complications × percentage with no infectious complications) + (cost per stay with infectious complications × percentage with at least one infectious complication). Both of these calculations allowed for a single patient to experience more than one infectious complication.

Average selling prices for immunonutrition were obtained from Nestlé HealthCare Nutrition. The costs for perioperative immunonutrition were calculated as follows: three servings of oral supplement for five days before surgery at $29 per day and one liter of enteral formula per day for seven days after surgery at $36 per day. The cost of standard nutrition was not considered in the analysis.

### Sensitivity analyses

Two sensitivity analyses were performed. First, for the combined upper and lower GI population, the infectious complication rate for the control group was allowed to vary from 0% to 40%, and the potential cost savings were estimated. Second, since the average length of stay is different for upper and lower GI cancer surgery and length of stay and infectious complication rates have been declining in the US with the adoption of less invasive surgical techniques, the potential cost savings were estimated using, for the control population, infectious complication rates and length of stay for the combined population and the two populations separately estimated from the 2008 HCUP NIS data rather than those from trials included in the meta-analysis. For both of these sensitivity analyses, Waitzberg and colleague’s [4] estimates of the percentage reduction in the infectious complication rate or length of stay of the control group associated with the use of perioperative immunonutrition was used to estimate the reduction in infectious complication rate or length of stay for the groups receiving immunonutrition.

### Institutional Review Board Consideration

RTI International’s Institutional Review Board (RTP, NC, USA) determined that this study met all criteria for exemption because only de-identified patient-level data or aggregated patient data were used in the analysis.

## Results

Results of the HCUP NIS analyses of costs are presented in Table 2. The NIS data demonstrated that, for all patients undergoing elective surgery for gastrointestinal cancer in the US in 2008, the mean cost per stay for patients presenting with infectious complications (or presenting with infectious plus other complications) was $53,361 ($41,119), whereas for those without infectious complications (or without infectious plus other complications) it was $20.406 ($19,628). The average cost per hospital day was $2,948. However, the average cost per day and costs per stay were higher for those undergoing upper GI cancer surgery than for those undergoing lower GI cancer surgery. Also presented in Table 2 is the average length of stay for the populations of interest. Of note are the lower average length of stay and infectious complication rates for the combined upper and lower GI cancer surgery patients than those observed in the clinical trials reviewed by Waitzberg and colleagues [4].

**Table 2:** **Estimated cost and length of stay with and without complications: results using 2008 HCUP NIS data**^a^

**Input variable**	**With complications (SD)**	**Without complications (SD)**	**With or without complications (SD)**
*Upper and lower GI combined, N = 95,198; Infectious Complication rate, 11.2%*			
Cost per day			$2,948 ($2,642)
Cost per stay	$53,361 ($62,039)	$20,406 ($21,541)	$24,096 ($30,838)
Length of stay	18.24 (16.25)	7.43 (4.99)	8.64 (7.96)
*Upper and Lower GI Combined, N = 95,198, Infectious + Other Complication Rate = 20.8%*			
Cost per day			$2,948 ($2,642)
Cost per stay	$41,119 ($52,420)	$19,628 ($19,580)	$24,096 ($30,838)
Length of stay	14.81 (13.41)	7.02 (4.49)	8.64 (7.96)
*Upper GI only, N = 11,291; Infectious Complication rate, 17.1%*			
Cost per day			$3,773 ($4,733)
Cost per stay	$85,451 ($89,124)	$34,194 ($37,964)	$42,895 ($53,988)
Length of stay	25.03 (19.92)	10.11 (6.32)	12.65 (12.61)
*Lower GI only, N = 83,974; Infectious Complication rate, 10.4%*			
Cost per day			$2,838 ($2,167)
Cost per stay	$46,320 ($51,728)	$18,697 ($17,764)	$21,572 ($25,147)
Length of stay in days	16.75 (14.48)	7.09 (4.42)	8.10 (6.93)

Abbreviations: *NIS*Â Nationwide Inpatient Sample; *GI* gastrointestinal; *HCUP* Healthcare Cost and Utilization Project; *SD* standard deviation.

^a^ Costs in flated from 2008 dollars to 2010 using the medical care component of the US Consumer Price Index.

When applying the HCUP NIS costs for hospital stays with and without infectious complications for the combined upper and lower GI population to the infectious complication rates with and without immunonutrition estimated in the Waitzberg meta-analysis, the mean cost per stay was $26,402 per patient in the control group and $22,743 per patient in the immunonutrition group, leading to $3,300 (rounded to the nearest $100) saved per patient due to immunonutrition use perioperatively attributable to the reduced complication rate. Using the estimated reduction in hospital length of stay of 2.18 days from Waitzberg’s meta-analysis gave an estimated reduction in cost of $6,000 (rounded to the nearest $100) per patient with immunonutrition when compared with standard nutritional supplementation (2.18*$2948)–$397. These results are presented in Table 3.

**Table 3:** Estimated cost savings per patient with the use of immunonutrition


	**Cost Savings per Patient by Costing Method**^**a**^	
**Population**	**Cost Savings Due to Reduced Hospital LOS**	**Cost Savings Due to Lower Infectious Complication Rates**
Base-case estimates		
Waitzberg et al. [4].	$6,000	$3,300
ΔLOS = 2.18; ICR, 14.71% vs. 31.32%		
Estimates using lower LOS and infectious complication rates for the control population^b^		
Upper + lower GI	$3,200	$1,600
ΔLOS = 1.21; ICR, 5.2% vs. 11.2%		
Upper GI only	$6,300	$4,300
ΔLOS = 1.78; ICR, 7.9% vs.17.1%		
Lower GI only	$2,800	$1,200
ΔLOS = 1.14; ICR, 4.8% vs. 10.4%		

Abbreviations: Δ, absolute difference; *GI* gastrointestinal; *HCUP* Healthcare Cost and Utilization Project; *ICR* infectious complication rate for perioperative immunonutrition vs. standard nutrition; *LOS* length of stay.

^a^ Rounded to the nearest $100.

^b^ Infectious complication rates and length of stay for the control population taken from 2008 HCUP NIS values presented in Table 2 and relative risk of infectious complications and percentage reduction in length of stay presented in Table 1 and taken from Waitzberg et al. [4].

**Figure 1:**
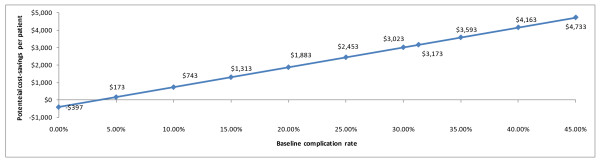
Cost savings attributable to reduced infectious complications (presented in 2010 US dollars).

The first sensitivity analysis aimed at determining the baseline infectious complication rate at which cost savings from use of immunonutrition would offset the cost of therapy. As illustrated in Figure 1, net cost savings continued to be observed for infectious complication rates above 3.5%.

In the second sensitivity analysis, the infectious complication rates, length of stay, and costs per day and per stay with and without infectious complications were taken from the 2008 HCUP NIS data analysis to show the impact on cost savings for the combined population as well as upper and lower GI cancer patients separately, using more recent data on length of stay and infectious complication rates. As presented in Table 3, the results show that the cost savings are lower when using the infectious complication rates and length of stay from the total HCUP NIS GI cancer surgery population and the lower GI cancer surgery population but higher for the upper GI cancer surgery population because of the higher hospital costs for the upper GI patients.

## Discussion

Multiple studies (>20), including those studies performed in patients undergoing elective surgery for gastrointestinal cancers, support the use of immunonutrition as a low-risk strategy that effectively and significantly decreases the risk of postsurgical complication in patients undergoing major elective surgery [4,5]. The analyses presented in this paper have demonstrated that the use of perioperative (both before and after surgery) immunonutrition also may be cost saving based on a meta-analysis of available trials in GI cancer patients using a single immunonutrition formula. Cost savings with the use of immunonutrition have been previously reported. However, the applicability of these studies has been limited due to the fact that their analysis is done on either a single clinical trial for perioperative [12] or preoperative immunonutrition [1,13] or of a hospital database for different patient populations [14].

The estimated net cost savings was calculated through two different approaches based on either a decrease in infectious complication rate or in hospital length of stay resulting from the use of immunonutrition. Interestingly, even though a significant decrease in cost was evident through either method, cost savings estimates based on decrease in the infectious complication rate were lower than estimates based on reduced hospital length of stay. This suggests that immunonutrition may bring additional benefits beyond reduction in risk of infectious complications, including a reduced risk of noninfectious complications (such as the reduction in anastomotic leak shown in the Waitzberg and colleagues [4] meta-analysis), decreased severity of complications (such as a more limited infection), or speedier recovery in patients who do not develop complications. Indeed, some clinical observations suggest that the use of immunonutrition results in improved hemodynamics, including decreased accumulation of lactate and base deficit, enhanced tissue oxygenation, and improved T-lymphocyte function [15]. These physiologic benefits may translate into clinical benefits that are difficult to quantify but nonetheless may result in a shorter length of stay.

Our results using the incremental costs associated with infectious complications were sensitive to the baseline infectious complication rate, suggesting that cost savings would decrease in patient populations or hospitals with lower infectious complication rates. However, cost savings are estimated with the use of immunonutrition when the infectious complication rate is above 3.5%.

Our study has some limitations. First, noninfectious complications were not included in the estimates that were based solely on estimated reductions in infectious complication rates, although the Waitzberg and colleagues [4] meta-analysis indicated a possible significant reduction in the noninfectious complication, anastomotic leak (Table 1). However, the estimates based on hospital length of stay would capture the effects of any reductions in noninfectious complication. Second, the studies included in the meta-analysis were completed over 10 years ago, and surgical techniques and postoperative management have evolved since then. Our sensitivity analysis using the 2008 HCUP NIS data was performed to assess the impact of more recent estimates of infectious complication rates and hospital length of stay in the US to address this limitation. Third, we used results from a meta-analysis published in 2006. However, more recent meta-analyses have estimated similar reduction in infectious complication rates and reductions in hospital length of the stay for those taking immunonutrition perioperatively for gastrointestinal surgery [5-9]. For example, the Marimuthu and colleagues [9] meta-analysis, which included data from several more recent clinical trials, estimated a relative risk (RR) for infectious complications of 0.53 and a reduction in hospital length of stay of 2.71 days for those receiving immunonutrition perioperatively. Their meta-analysis showed a lower impact on length of stay when only preoperative immunonutrition was provided (1.46 days), but a similar impact on length of stay when only postoperative immunonutrition was provided (2.44 days). Conversely, they showed a greater impact on infectious complication rates with only preoperative immunonutrition (RR = 0.48) and a lower impact with only postoperative immunonutrition (RR = 0.68) than that estimated for perioperative immunonutrition.

## Conclusions

Our study, which used pooled data from multiple clinical trials, demonstrates that a comparatively small investment in immunonutrition support both before and after surgery for both upper and lower gastrointestinal cancer surgery patients is likely to be more than offset by the estimated savings due to reduced infectious complications and/or hospital length of stay. Thus, the use of immunonutrition support may be a valuable addition to other strategies used by hospitals to control postsurgical infectious complications, improve patient outcomes, and reduce hospital costs.

## Abbreviations

CI: Confidence interval; Δ: Absolute difference; GI: Gastrointestinal; HCUP: Healthcare Cost and Utilization Project; ICD-9-CM: International Classification of Diseases; 9^th^ Revision: Clinical Modification; ICR: Infectious complication rate; LOS: Hospital length of stay; N: Number in sample; NIS: Nationwide Inpatient Sample; RR: Relative risk; SD: Standard deviation; US: United States.

## Competing interests

Funding support for the preparation of this manuscript was received from NESTLÉ Healthcare Nutrition (NESTLE), the maker of IMPACT®, an immunonutrition formula. two authors are employees of RTI Health Solutions and received funding from NESTLÉ to conduct this study and prepare this manuscript (JM and SC), and two authors are employees of NESTLÉ (HC-S and JO). JO is currently taking a leave of absence from his work as professor of surgery and critical care at the University of Pittsburgh.

## Authors’ contributions

SC was responsible for performing the cost analyses using the NIS data and for interpretation of the results and review of the manuscript; JM was responsible for study design and interpretation of results and for preparing the manuscript; HC-S and JO contributed to study design and interpretation of the results and to the writing and review of the manuscript. All authors had full access to all of the data in the study, all take responsibility for the integrity of the data and the accuracy of the data analysis. All authors have read and approved the final manuscript.
